# Compliance of postoperative instructions following the surgical extraction of impacted lower third molars: A randomized clinical trial

**DOI:** 10.4317/medoral.20121

**Published:** 2014-12-05

**Authors:** Joaquín Alvira-González, Cosme Gay-Escoda

**Affiliations:** 1 DDS, MS. Master degree program in Oral Surgery and Implantology. Faculty of Dentistry – University of Barcelona Spain; 2 MD, DDS, MS, PhD. Chairman and professor of Oral and Maxillofacial Surgery, Director of the Master Degree Program in Oral Surgery and Implantology, Faculty of Dentistry, University of Barcelona, Coordinator & Researcher of the “Fundació Institut d’Investigació Biomedica de Bellvitge” (IDIBELL Institute), L’Hospitalet de Llobregat, and Oral and Maxillofacial Surgery Departament, Hospital Quirón Teknon, Barcelona, Spain

## Abstract

Objectives: The understanding and adherence to postoperative care instructions are factors that influence the recuperation process after any surgical procedure. The aim of this study was to determine the percentage of patients who strictly follow the postoperative instructions after the extraction of an impacted lower third molar in relation to sociocultural level, preoperative anxiety scores and how postoperative information is provided to the patient. Study Design: Patients were randomly assigned to one of three different test groups according to how the postoperative instructions were presented: verbal, written and a group that received additional information. Before surgery, patients were required to complete the Corah Dental Anxiety Scale and personal information (age, gender and educational level) was also collected. *P*<0.05 was considered significant. Patients were surveyed a week after surgery regarding their adherence to postoperative instructions. Results: 84 patients (45 women and 39 men with an average of 28.23 ± 7.41 years) completed the study. There were no statistically significant differences regarding adherence of postoperative care instructions depending on the manner of instruction presentation, preoperative anxiety level and sociocultural level (*p*> 0.05). Quitting smoking or drinking of alcoholic/carbonated beverages were the main influential factors for the lack of adherence to postoperative care instructions during the week after surgery. Conclusions: Presentation of postoperative instructions, preoperative anxiety scores and sociocultural level do not appear to be key factors that promote the adherence to postoperative instructions.

** Key words:**Compliance, postoperative instructions, treatment, third molar.

## Introduction

Lower third molar extraction is the most common surgical procedure in Oral Surgery and it appears well documented in literature. Several authors have described different factors that may influence the degree of difficulty as well as the impact of this procedure on the quality of life of patients during the postoperative care (1-4). However, the postoperative period is also influenced by the understanding of the patient and the subsequent implementation of the guidelines presented by the professional in order to minimize morbidity, complications and to improve the quality of life of the patient.

The main elements that could interfere with the understanding of postoperative care instructions are how they are presented by the professional (verbally and/or written) and the sociocultural level of the patient (5-7). However, there are no references in the literature that consider how these two concepts may influence the adherence to postoperative instructions or its possible correlation to the level of preoperative anxiety that is common in any surgical procedure. All these factors could be obstacles to the proper adherence to postoperative instruction for the patient during the recovery period.

The main objective of this study was to determine the percentage of patients who strictly follow the postoperative instructions after the extraction of the impacted lower third molar, taking into account how the information is presented to the patient, the sociocultural level and level of preoperative anxiety. Secondly, to identify the main points of noncompliance and the reason for failure. The hypothesis is that patients with a lower level of preoperative anxiety, higher sociocultural level and who are given both full verbal and written information about the postoperative course will strictly follow postoperative instructions.

## Material and Methods

A total of 90 patients with an impacted lower third molar that required surgical removal for the first time were selected. The patients chosen for the study were healthy (ASA I) or had mild systemic disease without functional limitation (ASA II). All patients underwent surgical extraction of impacted lower third molar. Patients excluded from the study were those who could not attend the scheduled appointments, had a limited intelligence quotient, some psychological disorder or mental condition and had difficulties in language comprehension. The exclusion criteria ensured that only the patients that had no difficulties in understanding and following through with the study were selected.

The study was designed according to CONSORT guidelines for randomized clinical trials and was approved by the Research Ethics Committee (CEIC) of the Dental Clinic of the University of Barcelona, Spain (8). All patients were treated in the Hospital Odontològic de Bellvitge, University of Barcelona, Spain. The incorporation of each subject in the study was decided before knowing the assigned group. Patients were randomly assigned to one of the following three study groups (sequence generated by www.randomization.com).

• Verbal: postoperative instructions were given verbally together with a prescription sheet of the postoperative medication.

• Written: the usual postoperative instructions were given verbally and written, as well as the postoperative medication.

• Additional information: instructions and postoperative medication were given both verbally and written, and additional written information about the postoperative period was also provided ( Table 1, Table 2).

**Table 1:**
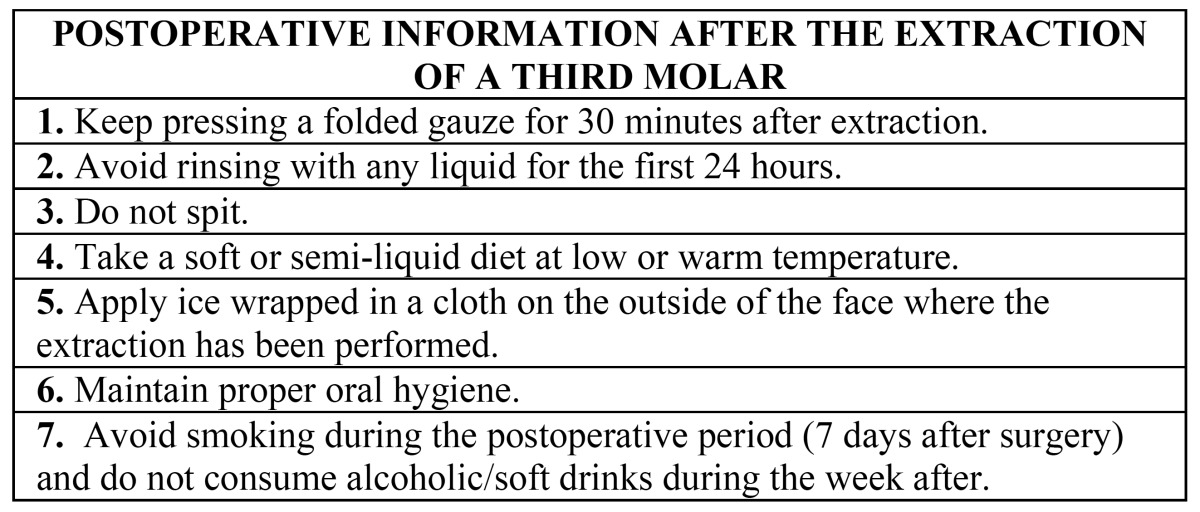
Postoperative information provided to written and additional information groups after the surgical extraction of third molars.

**Table 2:**
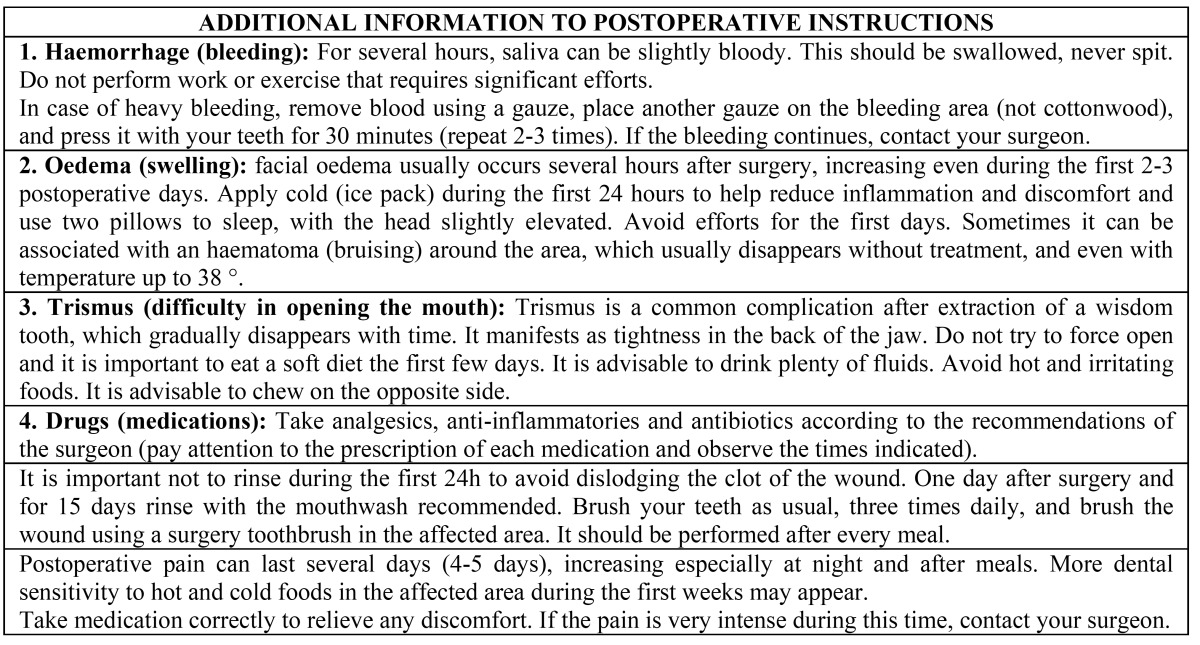
Information provided to the group of additional information only.

Patients were interviewed about their adherence to the instructions one week after the surgery, at the time of suture removal. They were requested about the compliment of the instructions given, number of days that medication and recommendations were followed and the reason of abandon ( Table 3).

**Table 3:**
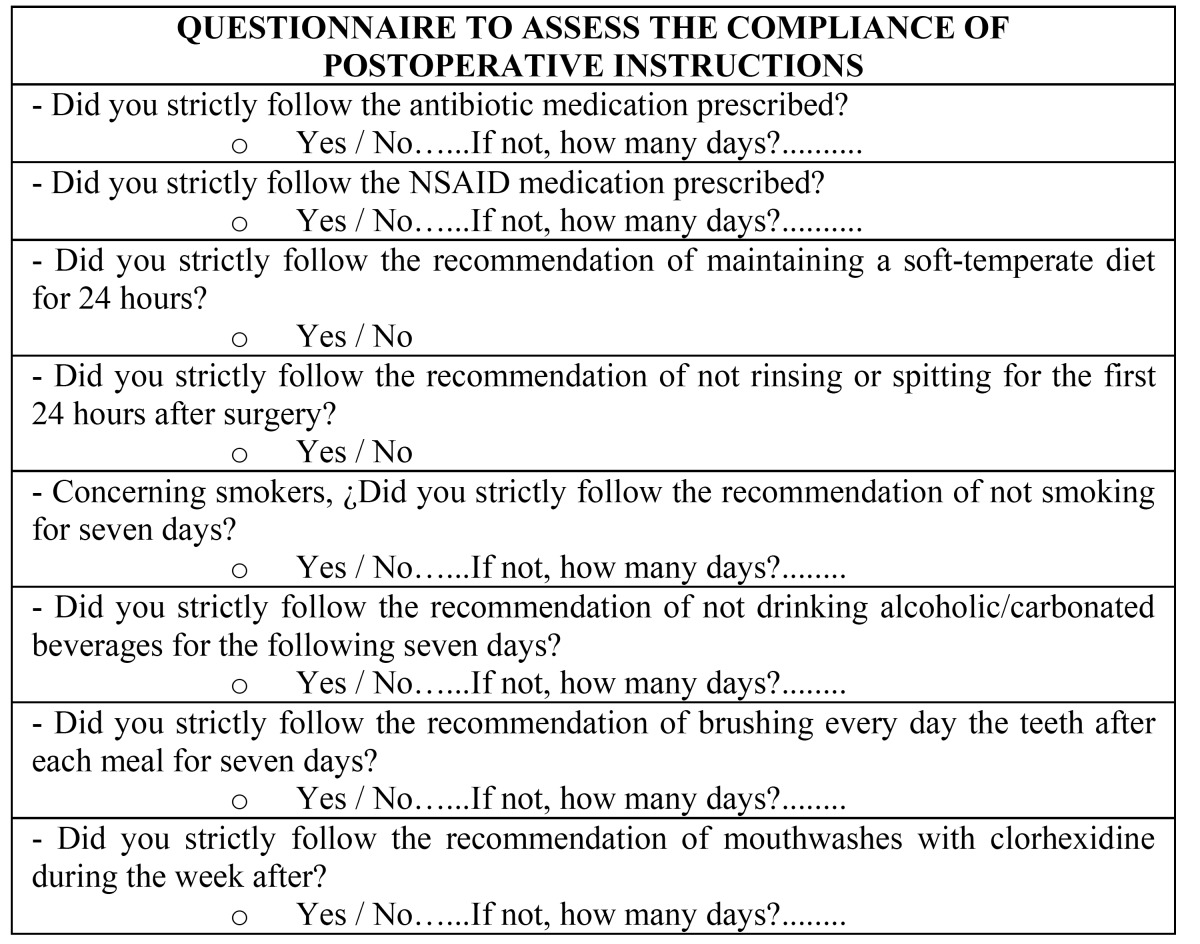
Questionnaire to assess the compliance of postoperative instructions following the surgical extraction of impacted lower third molars.

Before surgery, patients completed the Corah Dental Anxiety Scale (9) and data about age, gender and educational level (Basic, High School / Vocational Training, University) was also collected. Postoperative instructions were given by one resident previously instructed to provide similar instructions according to the study group to which the patient belonged. All surgical interventions were carried out by second year residents of the Master of Oral Surgery and Implantology (Barcelona University) with a similar surgical technique. Patients did not receive any financial compensation for their participation in the study.

The extraction of the impacted lower third molars was performed under local anaesthesia with articaine 4% and epinephrine 1:100.000 (Artinibsa, Inibsa, Lliça de Vall, Spain). The surgical area and all materials were steriles. The surgeon lifted a full-thickness flap that was protected by a cheek retractor. Lingual flap retraction with a Freer periostotom was performed only when deemed necessary by the surgeon. A sterile hand piece at low speed (20,000 rpm) and irrigation with sterile distilled water was used to do the ostectomy and the dental sectioning of the third molar, if needed. The wound was sutured with silk 3-0 (Silkam®, Braun, Tuttlingen, Germany). The surgical technique was similar to that described by Leonard (10). The following postoperative medication was prescribed.

• Antibiotic (750 mg Amoxicilin. Clamoxyl® -GlaxoSmithKline, Madrid, Spain) orally one tablet every 8 hours for 7 days).

• A NSAID (600mg Ibuprofeno. Espidifen® - Zambon, Barcelona, Spain) orally every 8 hours for 5 days).

• An analgesic (575 mg Metamizol. Nolotil® (Boehringer Ingelheim, Barcelona, Spain) orally, every 8 hours as relief medication).

• Mouthwash (Chlorhexidine Lacer® to 0.12 % (Lacer, Barcelona, Spain), the mouthwash twice daily for 15 days).

All surgeons involved in the study were blinded for which group each patient belonged. Statistical analysis was done using SPSS 15.0 for Windows (SPSS v15.0, SPSS Inc. Chicago, USA, licensed from the University of Barcelona). Demographic data was analysed using Chi-square test and ANOVA test. Chi-square test was used to compare the compliance according to preoperative anxiety level, sociocultural level and how the postoperative instructions were provided. The significance level was set at *p* <0.05.

## Results

**Figure 1:**
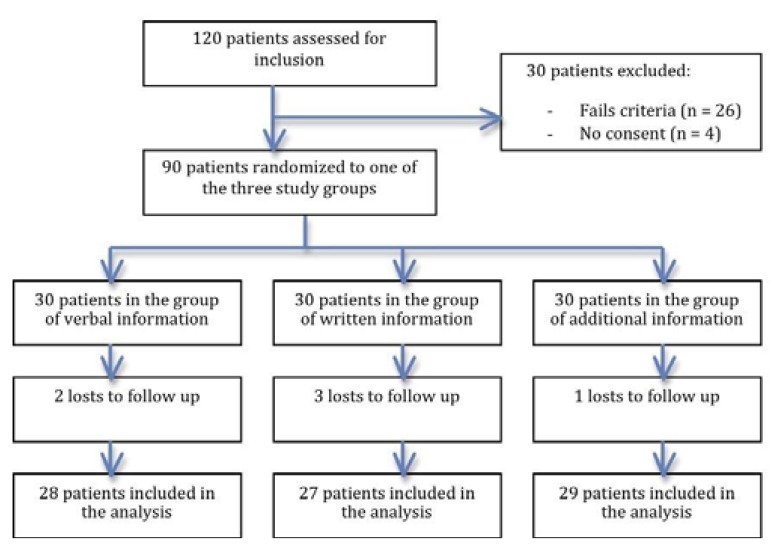
Flow chart of the participants in the trial.

Out of the 90 patients who began this study, six were eliminated because they did not attend the follow-up visit one week after surgery (Fig. 1). The results, therefore, are based on 84 patients (45 women and 39 men with an average age of 28.23 ± 7.41 years) distributed in different test groups according to how postoperative instructions were provided, sociocultural level and the level of preoperative anxiety.

- Type of information (verbal, written and additional)

The degree of adherence to the postoperative guidelines provided by the professional after surgery is shown in  Table 4. Stopping smoking for the following seven days was one of the guidelines less followed by patients (only 45.6% of compliance). This was followed by the consumption of alcohol/carbonated beverages (80.9%). There were no statistically significant differences in the adherence to postoperative instructions regarding how the instructions were provided to the patients ( Table 4), without differences between groups in terms of gender (*p*=0.627), age (*p*=0.840), sociocultural level (*p*=0.790) or preoperative anxiety (*p*=0.914).

**Table 4:**
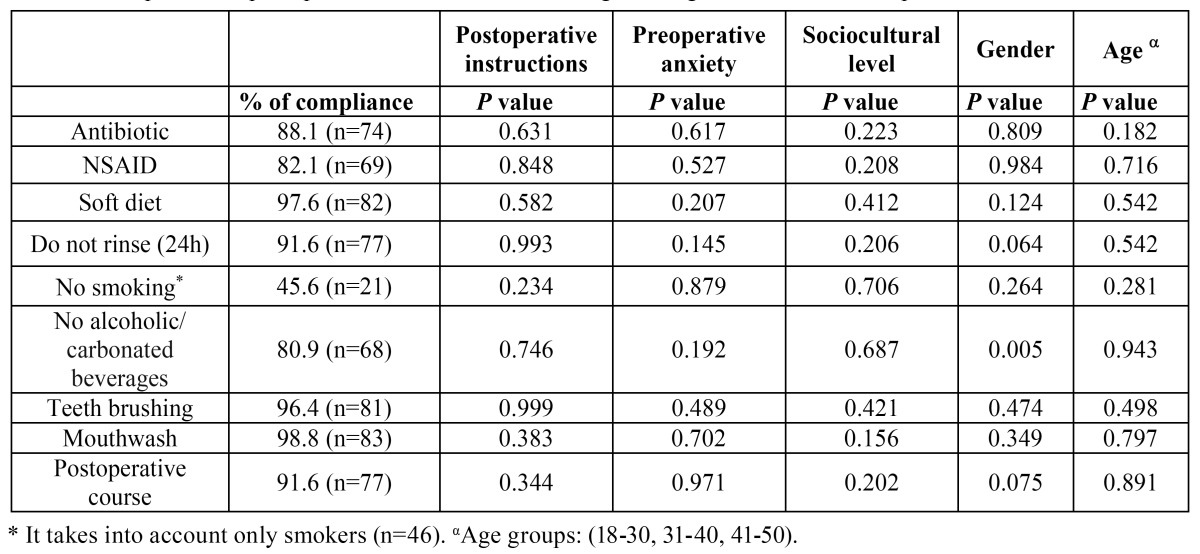
Compliance of postoperative instructions following the surgical extraction of impacted lower third molars.

- Preoperative Anxiety

**Figure 2:**
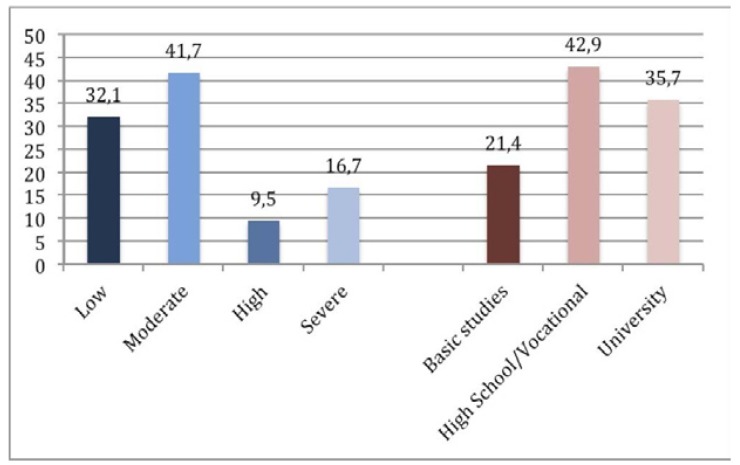
Distribution of the percentages of patients according to the level of preoperative anxiety (blue) and educational level (red).

Approximately 74% of patients reported low or moderate anxiety when facing the first surgical removal of a third molar (Fig. 2). A greater level of anxiety recorded preoperatively did not lead to a greater degree of non-compliance ( Table 4), during the seven days after surgery (*p* >0.05).

- Sociocultural level, gender and age

There were no statistically significant differences in following postoperative instructions according to sociocultural level or age ( Table 4 and Fig. 2). In addition, gender is not a factor associated with a greater non-adherence to postoperative instructions, with exception of following the guidelines that prohibit the consumption of alcoholic/carbonated beverages within the seven days after surgery. Approximately 43.5% of men failed to follow it. Most of the patients (91.6%) found that the postoperative course experienced was very similar to that explained on the day of surgery, regardless sociocultural level and how the postoperative instructions were provided.

## Discussion

The understanding and the subsequent implementation of postoperative guidelines are factors that influence the recovery from any surgical procedure. Some authors state that instructing patients about postoperative care not only reduces postoperative morbidity, but also improves the quality of life during recovery period (5,11-13). However, there are a number of variables that may interfere with the extent and quality of information. For example, how the information is presented, to provide additional information and whether factors such as the level of preoperative anxiety, sociocultural level and age have a role in the adherence and complete understanding of postoperative guidelines.

The non-adherence to postoperative instructions was principally caused by failure of quitting smoking and/or the consumption of alcoholic/carbonated beverages during the week after surgery, followed by not taking correctly the prescribed medicine (antibiotics and anti-inflammatory medication), also observed by Blinder *et al*. (14). Tobacco represented the main cause of noncompliance, with over 50% of smokers that continued smoking during the seven days after surgery, regardless of gender, age, level of preoperative anxiety, sociocultural level or how to provide postoperative information. Grossi *et al*. (15) and Heng *et al*. (16) identified the smoking habit as a contributing factor of increased postoperative complications and worse postoperative course after a tooth extraction. Consumption of alcohol and carbonated beverages during the postoperative period was biased by gender, mainly associated with the male gender.

The improper use of antibiotic medication carried out by patients is primarily due to popular beliefs and ignorance about the prescribed medication (17). Pain relief was the main cause of abandonment of anti-inflammatory and antibiotic prescription in our study (40% and 53.3% respectively), without differences between gender, age, level of preoperative anxiety, sociocultural level or how to provide postoperative information. However, Culbertson *et al*. (18) pointed out that more than half of the patients in their study preferred both, verbal and written information, about the medication that was prescribed to them. Orero-González *et al*. (17) found that 32% of patients self-medicate in the Spanish population, those being oral and respiratory infections the main cause of self-medication. As Blinder *et al*. (14) advised, it is important to explain properly the need to prescribe and inform the patient of the usefulness of antibiotics because self-medication or a period of inappropriate use of antibiotics can be harmful to the health of the patient.

Vallerand *et al*. (5) showed that providing postoperative instructions both verbal and written improved compliance of the instructions given by the professional after third molar removal. Houts *et al*. (19) stated that patients remembered only 14% of the information when given verbally, compared to 80% when combined with pictograms, while some authors found that verbal instructions alone were ineffective (6,14,20). In our study, there were not statistical differences in terms of adherence to postoperative guidelines between the groups regarding how the information was presented to the patient. In addition, most patients reported that the postoperative course they experienced did not differ from the information given after the surgical intervention, regardless of the method of presenting the postoperative instructions.

As indicated by Kessels (7), patients forget between 40%-80% of the data given by the professional almost immediately, being the sociocultural level and the age influential factors in comprehension and implementation of postoperative instructions. Besides, there are other closely related variables that must be taken into account and could influence in a suitable patient-professional interaction. Alexander (6) and Kessels (7) emphasize that the degree of illiteracy and ignorance of the language are crucial elements in understanding and compliance of the instructions. There were no statistically significant differences in compliance between different population groups regarding age and educational level in our study. Due to the fact that other variables (illiteracy or ignorance of the language) have not been taken into account in our study group, this could have generated some selection bias in the studied population.

The anxiety generated by any unknown experience is not only a factor related to higher levels of pain perception, but also represents an obstacle for the patients, limiting their attention and compliance of any postoperative instructions given after surgery, especially if they are verbal (5,7,20-23). Besides, as indicated by Brasileiro *et al*. (24) in their study, patients with previous experience of extractions brought up different questions about the procedure and showed lower anxiety levels compared to patients who have not experienced an extraction. In our study we found that higher levels of preoperative anxiety did not affect the adherence to postoperative instructions after the first surgical extraction of a third molar.

Although several variables could interfere with the adherence to postoperative guidelines, Alexander (6) suggests adapting the instructions to the needs of each patient or case (especially with regard to the limitations of understanding certain terms and ignorance of the language), because a complete and detailed postoperative course and postoperative instructions not only reduces the anxiety that a patient would experience, but also encourages adherence to them indirectly.

## Conclusions

Smoking and the consumption of alcoholic/carbonated beverages during the week after surgery represents the main factors of noncompliance after the extraction of a lower third molar, the latter associated with the male gender. Despite that no differences were found in the compliance of postoperative instructions between the groups regarding the presentation of postoperative instructions, the sociocultural level or the level of preoperative anxiety, it is essential to meet the needs of each patient and provide full details of the postoperative course as well as postoperative instructions.
